# Histogram analysis of diffusion kurtosis imaging of nasopharyngeal carcinoma: Correlation between quantitative parameters and clinical stage

**DOI:** 10.18632/oncotarget.17591

**Published:** 2017-05-03

**Authors:** Xiao-Quan Xu, Gao Ma, Yan-Jun Wang, Hao Hu, Guo-Yi Su, Hai-Bin Shi, Fei-Yun Wu

**Affiliations:** ^1^ Department of Radiology, The First Affiliated Hospital of Nanjing Medical University, Nanjing, China

**Keywords:** nasopharyngeal carcinoma, clinical stage, magnetic resonance imaging, diffusion kurtosis imaging, histogram

## Abstract

**Purpose:**

To evaluate the correlation between histogram parameters derived from diffusion-kurtosis (DK) imaging and the clinical stage of nasopharyngeal carcinoma (NPC).

**Results:**

High T-stage (T3/4) NPC showed significantly higher K_app-mean_ (*P* = 0.018), K_app-median_ (*P* = 0.029) and K_app-90th_ (*P* = 0.003) than low T-stage (T1/2) NPC. High N-stage NPC (N2/3) showed significantly lower Dapp-mean (*P* = 0.002), Dapp-median (*P* = 0.002) and Dapp-10th (*P* < 0.001) than low N-stage NPC (N0/1). High AJCC-stage NPC (III/IV) showed significantly lower D_app-10th_ (*P* = 0.038) than low AJCC-stage NPC (I/II). ROC analyses indicated that Kapp-90th was optimal for predicting high T-stage (AUC, 0.759; sensitivity, 0.842; specificity, 0.607), while Dapp-10th was best for predicting high N- and AJCC-stage (N-stage, AUC, 0.841; sensitivity, 0.875; specificity, 0.807; AJCC-stage, AUC, 0.671; sensitivity, 0.800; specificity, 0.588).

**Materials and Methods:**

DK imaging data of forty-seven consecutive NPC patients were retrospectively analyzed. Apparent diffusion for Gaussian distribution (D_*app*_) and apparent kurtosis coefficient (K_*app*_) were generated using diffusion-kurtosis model. Histogram parameters, including mean, median, 10th, 90th percentiles, skewness and kurtosis of D_*app*_ and K_*app*_ were calculated. Patients were divided into low and high T, N and clinical stage based on American Joint Committee on Cancer (AJCC) staging system. Differences of histogram parameters between low and high T, N and AJCC stages were compared using *t* test. Multiple receiver operating characteristic (ROC) curves were used to determine and compare the value of significant parameters in predicting high T, N and AJCC stage, respectively.

**Conclusions:**

DK imaging-derived parameters correlated well with clinical stage of NPC, therefore could serve as an adjunctive imaging technique for evaluating NPC.

## INTRODUCTION

Nasopharyngeal Carcinoma (NPC) is one of the most common head and neck malignancies in southern China and Southeast Asia [1]. Accurate pre-treatment staging is critical for clinicians to carry out individual treatment. Radiotherapy is the most common treatment for early-stage patients, while combination of neoadjuvant chemotherapy would benefit the patients in advanced-stage [2, 3]. Conventional magnetic resonance (MR) imaging demonstrates excellent diagnostic performance in identifying the T stage, however, diagnostic dilemma exists in the accurate identification of the nodal metastasis (N stage) and distant metastasis (M stage) [3]. This situation may be attributed to the fact that conventional MR imaging provide little functional information of the tumors.

Various functional MR imaging modalities have been proven to be useful to detect the micro-structure changes before the morphological changes appear. Among these, diffusion-weighted (DW) imaging and its derived apparent diffusion coefficient (ADC) have been proven to be useful in identifying the nodal status of NPC patients. Razek et al found that the ADC value of the NPC lesion was significantly lower when the metastatic lymph nodes were present [4]. The premise of DWI is that water molecule diffusion occurs in a free and unrestricted environment with a Gaussian distribution [5]. However, the complexity of the microstructural environment (high viscosity and cell membranes) can remarkably restrict water diffusion and lead to water diffusion deviating from Gaussian diffusion [6].

Recently, an emerging DW imaging technique, diffusion-kurtosis (DK) imaging which was firstly described by Jensen et al in 2005, has attracted more and more attention [7–9]. This model includes the calculation of kurtosis and diffusion coefficients. Kurtosis provides a more accurate quantification of the deviation of tissue diffusion from a Gaussian distribution. Diffusivity is the diffusion coefficient with correction of non-Gaussian bias [10]. Previously, Chen et al applied the DK imaging in the pre-treatment evaluation of the NPC, and found that early change of DK imaging-derived parameters was useful to predict the treatment response to neoadjuvant chemotherapy [11]. However, the relationship between DK imaging-derived parameters and the clinical stage of the NPC patients has not be clarified till now.

Moreover, regional region-of-interest (ROI) selection methods on one or several selected sections of the tumor were preferred in previous studies [12]. Such ROI selection methods may lead to the inter-reader variability and the inaccurate reflection of the tumor heterogeneity, which has been pointed out as a limitation of many studies [12]. By contrast, a histogram analysis approach encompassing the whole tumor tissue has been proven as a better way for differentiating benign from malignant tumors, and discriminating the pathological grade of various malignant tumors [12–14].

Therefore, the aim of our study was to evaluate the correlation between the DK imaging-derived histogram parameters and the clinical stages of NPC patients. We hypothesise that the DK imaging-derived histogram parameters can help to reflect the overall pre-treatment stage, and further serve as an adjunctive imaging technique for evaluating NPC.

## RESULTS

### T stage

**Figure 1:**
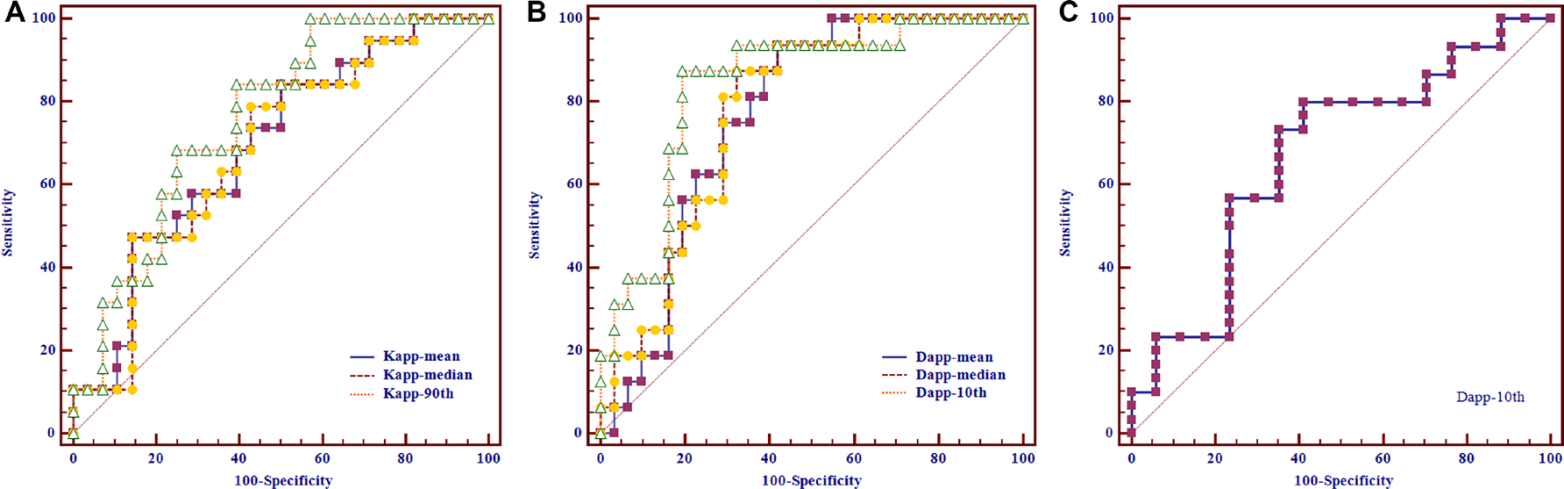
Receiver operating characteristic curves of using diffusion kurtosis imaging-derived histogram parameters to predict the nasopharyngeal carcinoma patients in high T-, N- and AJCC-stage, respectively (**A**) Setting K_*app-90th*_ as the differentiating index, optimal performance could be achieved in predicting high T-stage [cut-off value, 1.513; area under curve (AUC), 0.759; sensitivity, 0.842; specificity, 0.607]. (**B**) Optimal performance could be obtained when using D_*app-10th*_ as the differentiating index to predict high N-stage (cut-off value, 0.798 × 10^–3^ mm^2^/s; AUC, 0.841; sensitivity, 0.875; specificity, 0.807). (**C**) Setting D_*app-10th*_ = 0.862 × 10^–3^ mm^2^/s as the cut-off value, optimal performance (AUC, 0.671; sensitivity, 0.800; specificity, 0.588) could be obtained in predicting high AJCC-stage.

The mean volume of contoured tumors was 16.383 ± 18.341 cm^3^ (range from 1.278 to 81.849 cm^3^). Table 1 showed the comparisons of DK imaging-derived histogram parameters between low T-stage and high T-stage NPC. High T-stage NPC showed significantly higher K_*app-mean*_ (*P* = 0.018), K_*app-median*_ (*P* = 0.029) and K_*app-90th*_ (*P* = 0.003) than the low T-stage NPC, while no significant difference was found on K_*app-10th*_, skewness, kurtosis and all the D_*app*_ related histogram parameters (all Ps > 0.05). ROC analyses results showed that, setting K_*app-90th*_ = 1.513 as the cut-off value, optimal diagnostic performance (AUC, 0.759; sensitivity, 0.842; specificity, 0.607) could be obtained in predicting high T-stage (Table 2). The AUC of the ROC curve of K_*app-90th*_ was significantly higher than those of the K_*app-mean*_ (*P* = 0.049) and K_*app-median*_ (*P* = 0.037), while no significant difference between K_*app-mean*_ and K_*app-median*_ (*P* = 0.810) (Figure 1A).

**Table 1:** Comparison of diffusion kurtosis imaging derived parameters among different clinical stages

Parameter	T stage		P	N stage		P	AJCC stage		P
	T1 + T2	T3 + T4		N0 + N1	N2 + N3		I + II	III + IV	
Histogram D_app_									
D_app-mean_	0.954 ± 0.114	0.965 ± 0.102	0.748	0.992 ± 0.110	0.893 ± 0.070	0.002	0.974 ± 0.123	0.949 ± 0.101	0.460
D_app-median_	0.941 ± 0.117	0.950 ± 0.104	0.804	0.980 ± 0.111	0.876 ± 0.074	0.002	0.962 ± 0.123	0.934 ± 0.104	0.418
D_app-10th_	0.818 ± 0.099	0.789 ± 0.093	0.310	0.844 ± 0.086	0.733 ± 0.072	< 0.001	0.845 ± 0.099	0.784 ± 0.090	0.038
D_app-90th_	1.139 ± 0.216	1.167 ± 0.140	0.625	1.188 ± 0.213	1.077 ± 0.093	0.054	1.170 ± 0.264	1.139 ± 0.131	0.590
Skewness	0.399 ± 0.514	0.499 ± 0.395	0.478	0.447 ± 0.450	0.424 ± 0.514	0.870	0.420 ± 0.519	0.455 ± 0.446	0.810
Kurtosis	3.122 ± 1.197	3.359 ± 1.007	0.483	3.260 ± 1.304	3.135 ± 0.651	0.719	3.194 ± 1.450	3.240 ± 0.902	0.893
Histogram K_app_									
K_app-mean_	1.307 ± 0.165	1.417 ± 0.131	0.018	1.342 ± 0.170	1.350 ± 0.143	0.884	1.313 ± 0.125	1.363 ± 0.176	0.308
K_app-median_	1.320 ± 0.168	1.423 ± 0.128	0.029	1.350 ± 0.168	1.366 ± 0.146	0.747	1.324 ± 0.126	1.373 ± 0.175	0.318
K_app-10th_	1.116 ± 0.166	1.203 ± 0.158	0.080	1.141 ± 0.180	1.152 ± 0.143	0.837	1.125 ± 0.125	1.155 ± 0.185	0.554
K_app-90th_	1.476 ± 0.172	1.625 ± 0.135	0.003	1.532 ± 0.183	1.524 ± 0.151	0.879	1.487 ± 0.139	1.554 ± 0.185	0.203
Skewness	−0.367 ± 0.400	−0.491 ± 0.490	0.346	−0.369 ± 0.443	−0.512 ± 0.426	0.296	−0.279 ± 0.335	−0.531 ± 0.478	0.061
Kurtosis	3.377 ± 1.075	3.857 ± 0.963	0.124	3.581 ± 1.072	3.551 ± 1.033	0.928	3.271 ± 1.019	3.782 ± 1.021	0.106

Note: Data are reported as mean ± standard deviation; Unit for D_*app*_ related parameters is ×10^−3^ mm^2^/s. AJCC indicates American Joint Committee on Cancer.

**Table 2:** Diagnostic performance of diffusion kurtosis imaging derived parameters in predicting the high T, N and clinical stage

Parameter	Cut-off value	AUC	Sensitivity	Specificity
T stage				
K_*app-mean*_	1.275	0.692 (0.540–0.818)	0.842 (0.604–0.966)	0.500 (0.306–0.694)
K_*app-median*_	1.325	0.688 (0.536–0.815)	0.790 (0.544–0.939)	0.571 (0.372–0.755)
K_*app-90th*_	1.513	0.759 (0.613–0.872)	0.842 (0.604–0.966)	0.607 (0.406–0.785)
N stage				
D_*app-mean*_	0.968	0.766 (0.620–0.877)	0.938 (0.698–0.998)	0.581 (0.391–0.755)
D_*app-median*_	0.931	0.776 (0.631–0885)	0.875 (0.617–0.984)	0.677 (0.486–0.833)
D_*app-10th*_	0.798	0.841 (0.705–0.931)	0.875 (0.617–0.984)	0.807 (0.625–0.925)
AJCC stage				
D_*app-10th*_	0.862	0.671 (0.518–0.801)	0.800(0.614–0.923)	0.588 (0.329–0.816)

Note: Numbers in the parentheses are the 95% confidence intervals. Unit for D_*app*_ related parameters is ×10^–3^ mm^2^/s. AUC indicates area under curve; AJCC, American Joint Committee on Cancer.

### N stage

The comparisons of DK imaging-derived histogram parameters between low and high N-stage NPC were also summarized in Table 1. High N-stage NPC showed significantly lower D_*app-mean*_ (*P* = 0.002), D_*app-median*_ (*P* = 0.002) and D_*app-10th*_ (*P* < 0.001) than the low N-stage NPC, while no significant difference was found on D_*app-90th*_, skewness, kurtosis and all the K_*app*_ related histogram parameters (all Ps > 0.05). Setting D_*app-10th*_ = 0.798 × 10^–3^ mm^2^/s as the cut-off value, optimal diagnostic performance (AUC, 0.841; sensitivity, 0.875; specificity, 0.807) could be obtained in predicting high N-stage (Table 2). The AUC of the ROC curve of D_*app-10th*_ was significantly higher than those of the D_*app-mean*_ (*P* = 0.035) and D_*app-median*_ (*P* = 0.043), while no significant difference between D_*app-mean*_ and D_*app-median*_ (*P* = 0.535) (Figure 1B).

### AJCC stage

**Figure 2:**
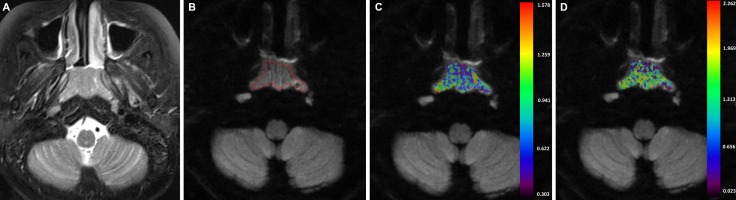
A-43-years-old man with nasopharyngeal carcinoma staged at T1N2M0 (**A**) Axial T2-weighted image showed a local mass which located in the nasopharynx region. (**B**) After region of interest was drawn around the mass, color maps for D_*app*_ (**C**) and K_*app*_ (**D**) were obtained. The D_*app-10th*_ and K_*app-90th*_ value of the mass were 0.629× 10^–3^ mm^2^/s and 1.562.

**Figure 3:**
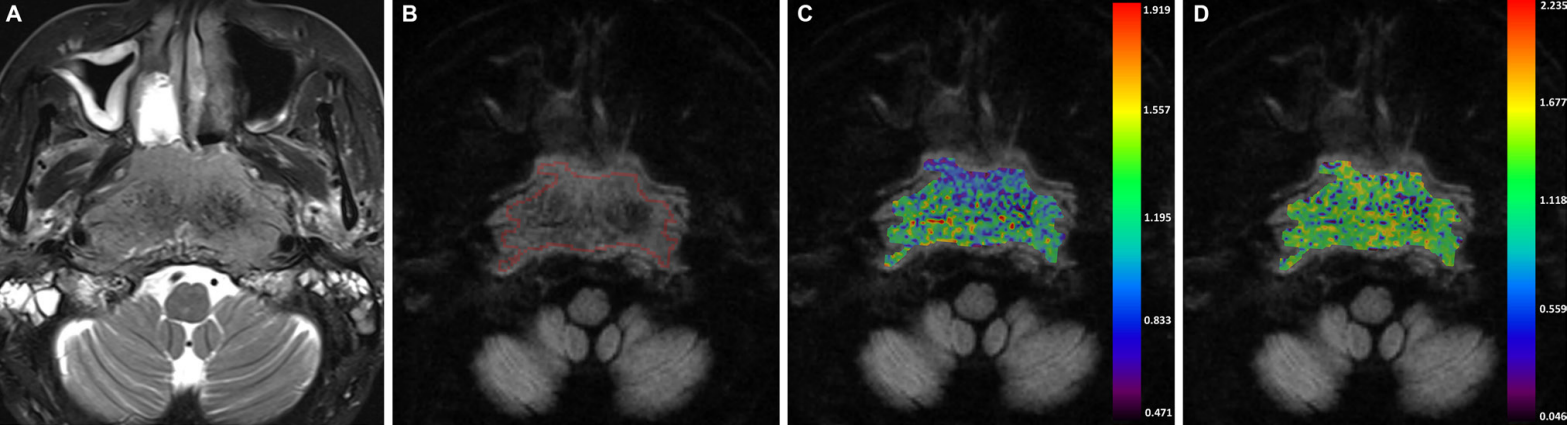
A-54-years-old man with nasopharyngeal carcinoma staged at T4N0M0 (**A**) Axial T2-weighted image showed an infiltrative mass that located in the nasopharynx region. (**B**) After region of interest was drawn around the mass, color maps for D_*app*_ (**C**) and K_*app*_ (**D**) were obtained. The D_*app-10th*_ and K_*app-90th*_ value of the mass were 0.828 × 10^–3^ mm^2^/s and 1.898.

**Figure 4:**
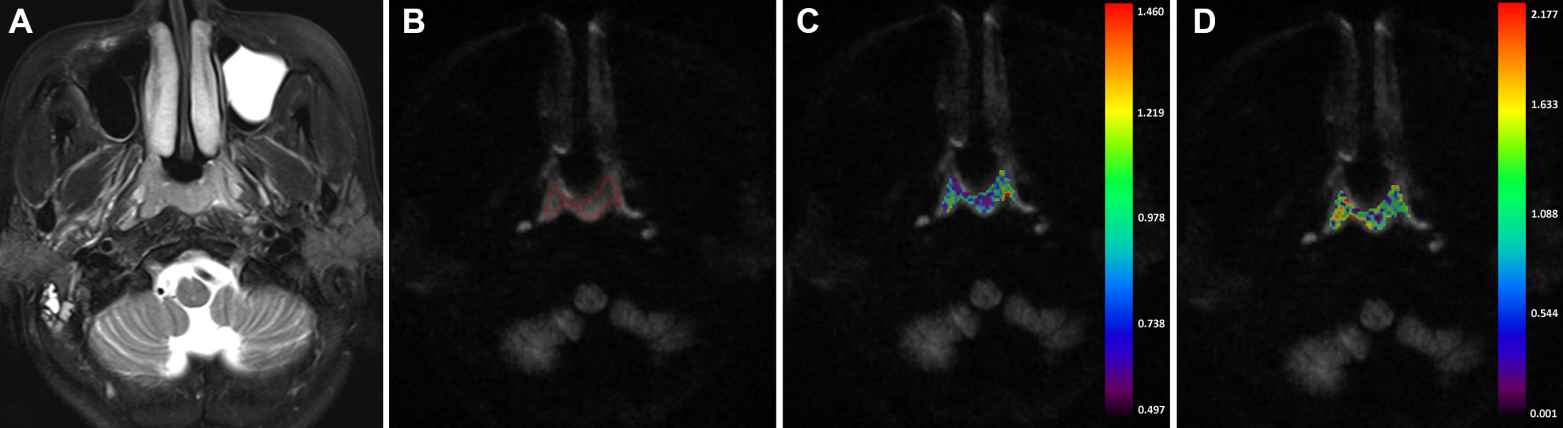
A-56-year-old man nasopharyngeal carcinoma staged at T1N2M0 (**A**) Axial T2-weighted image showed a small mass. (**B**) No obvious artifact was found on the DK image. After region of interest was drawn around the mass, color maps for D_*app*_ (**C**) and K_*app*_ (**D**) were obtained. The D_*app-10th*_ and K_*app-90th*_ value of the mass were 0.656× 10^–3^ mm^2^/s and 1.415.

Table 1 showed that the high AJCC-stage NPC demonstrated significantly lower D_*app-10th*_ (*P* = 0.038) than the low AJCC-stage NPC, while no significant difference was found on D_*app-mean*_, D_*app-median*_, D_*app-90th*_, skewness, kurtosis and all the K_*app*_ related histogram parameters (all Ps > 0.05). Table 2 showed that, setting D_*app-10th*_ = 0.862 × 10^–3^ mm^2^/s as the cut-off value, optimal diagnostic performance (AUC, 0.671; sensitivity, 0.800; specificity, 0.588) could be obtained in predicting high AJCC-stage (Figure 1C). The MR images of representative NPC patients are showed in Figures 2, 3, 4.

### Inter-reader agreement

Table 3 summarized the inter-reader agreements of the measurements by using the ICC. Good to excellent agreements were obtained for the measurements of DK imaging-derived histogram parameters (ICC, ranged from 0.621 to 0.961). Among them, inter-reader agreement was highest for the measurement of K_*app-median*_ (ICC, 0.961).

**Table 3:** Inter-reader intraclass correlation coefficient for the measurements of diffusion kurtosis imaging-derived histogram parameters

Parameters	Inter–reader ICC
Histogram D_app_	
D_*app-mean*_	0.952 (0.913–0.973)
D_*app-median*_	0.959 (0.926–0.977)
D_*app-10th*_	0.896 (0.814–0.942)
D_*app-90th*_	0.931 (0.875–0.961)
Skewness	0.621 (0.319–0.789)
Kurtosis	0.693 (0.449–0.829)
Histogram K_app_	
K_*app-mean*_	0.956 (0.920–0.975)
K_*app-median*_	0.961 (0.930–0.978)
K_*app-10th*_	0.881 (0.787–0.934)
K_*app-90th*_	0.941 (0.894–0.967)
Skewness	0.649 (0.369–0.804)
Kurtosis	0.765 (0.578–0.869)

Note: Numbers in the parentheses are the 95% confidence intervals. ICC indicates intraclass correlation coefficient.

## DISCUSSION

Accurate pre-treatment staging of NPC has been proven to be crucial for establishing the treatment strategy and predicting disease prognosis [3]. As an emerging imaging technique, DK imaging has been applied to study the heterogeneity and complexity of various tumors [5–7, 9–12]. However, the correlation between DK imaging-derived parameters and the clinical stage of the NPC patients has not been clarified till now. Our study firstly investigated this correlation, and found that D_*app-10th*_ was the optimal parameter to predict the high N- and AJCC-stage, while K_*app-90th*_ was promising to predict the advanced T-stage. Our results suggested that the DK imaging-derived parameters could potentially help the pre-treatment staging and treatment stratification of the NPC patients.

The K_*app*_ reflected the deviation from the Gaussion diffusion distribution. It increases with the microstructural complexity of the diseased tissue [11, 15]. In the field head and neck imaging, Jiang et al found that K value was significantly higher in malignant sinonasal lesions than in benign lesions [16]. This difference reflected the higher microstructural complexity which was usually found in malignant solid lesions. As to the relationship between K parameters and the tumoral T stage, Yu et al found that the K_app_ histogram parameters correlated well with histological T stage in the patients with rectal cancer [7]. Our study hold the similar findings, K_*app*_ of the high T-stage NPC was higher than that of the low T-stage NPC. K_*app*_ increased as the T-stage of the NPC increased. As we know, the NPC lesion in the advanced stage is prone to intra-tumoral necrosis [3]. The intra-tumoral necrosis would increase the complexity and heterogeneity of the advanced-stage NPC tissue, and naturally the high-T stage NPC lesions would demonstrate higher K_app_ value.

The status of cervical lymph nodes was an important predictor of the prognosis in patients with NPC. Prognosis worsened with an increased number of affected lymph nodes [4]. Our study indicated that the D_*app*_ of the NPC in high N-stage was lower than those in low N-stage. Similar association between lower diffusion value and the high N-stage in NPC has been reported previously [3]. Lai et al reported that the D value derived from intravoxel incoherent motion (IVIM) MR imaging of the high N-stage NPC was lower than that of the low N-stage NPC [3]. Considering the existed dilemma when using conventional MR imaging to assess the N-stage of NPC patients, we thought that our findings deserved great interest. Based on our findings, we suggested some functional MR imaging techniques, such as DK imaging or IVIM MR imaging, should also be applied in the pre-treatment evaluation of NPC patients. The derived functional information could provide supplementary information to the nodal size alone for accurate N stage.

In present study, D_*app-10th*_ was optimal for AJCC staging. High AJCC-stage NPC demonstrated lower D_*app-10th*_, and a cut-off value of 0.862 × 10^–3^ mm^2^/s was able to predict the high AJCC-stage with optimal diagnostic ability. This finding of a low diffusion value in a high AJCC-stage NPC group was also in accordance with the results from a recent IVIM MR study by Lai et al [3]. And they found that a D value of 0.782 × 10^–3^ mm^2^/s was the optimal cut-off value for differentiating high and low AJCC-stage NPC. The same point between our study and the study of Lai et al was the conclusion that diffusion metrics were associated with the AJCC stage of NPC patients. However, there were several different points between two studies, including the different fitting model and choice of b values. In the study of Lai et al, they acquired IVIM imaging data with 13b values (from 0–1000 s/mm^2^) and then processed them using bi-exponential model. However, we acquire DK imaging data with 4b values (including 1500 s/mm^2^) and processed them under the assumption of the No-Gaussian diffusion of water molecule. These different points might lead to the discordance of the cut-off value between two studies. In addition, we all know that the NPC patients were prone to lymph node metastasis. When the primary tumor was even very small, bilateral lymph node metastasis might have occurred. Therefore in our opinion, the clinical stage of NPC patients is more easily influenced by the N stage. Then, it was not surprising that diffusion parameter correlated well with AJCC stage, while kurtosis parameter did not.

In previous studies, quantitation of DW imaging was most commonly achieved based on the ROIs placed on one or several representative slice of the tumor [12, 17]. This method was subjective and prone to sampling bias, and this problem has been pointed out as a drawback of several studies. By contrast, a whole-tumor histogram approach could effectively eliminate the sampling bias and reflect the heterogeneity of the tumor tissue [12–14, 17, 18]. In the head and neck related imaging studies, the superiority of whole-tumor histogram approach has been proven in differentiating the histological grades of head and neck squamous cell carcinoma [19], discriminating orbital benign and malignant tumors [13] and so on. Therefore, we chose whole-tumor histogram approach in our study. Our results indicated that D_*app-10th*_ performed better than D_*app-mean*_ in predicting high N-stage, meanwhile K_*app-90th*_ was superior to K_*app-mean*_ in predicting high T-stage, which also confirmed the superiority of whole-tumor histogram approach.

In addition, nasopharynx is a technically difficult region to acquire high-quality DW imaging because of the neighboring air-containing structures and respiratory or swallowing motion artifacts [11]. Currently, DW imaging was mostly performed based on the single-shot echo-planar imaging, which is prone to image artifacts [21]. As an alternative approach, readout-segmented echo-planar imaging (RS-EPI) has been proven to be useful to reduce the geometric distortions, image blurring and ghosting artifacts in head and neck regions [20–22]. Therefore, we used RS-EPI technique for the DK imaging of nasopharynx in present study, we believed that the DK imaging-derived parameters based on RS-EPI could reflect the characteristic of the NPC more accurately.

Besides the nature of a retrospective study and the limited sample size, our study had several other limitations. First, because of the limited study period and follow-up time, we could not correlate the DK imaging-derived parameters with the patient outcome. Further study that analyze the relationship between DK imaging-derived parameters and the prognosis of NPC patients would be more clinically useful. Second, the slice thickness of conventional MR sequence and DK imaging was 4 mm, which would be too thick for the evaluation of small NPC lesions. Third, we did not correlate the DK imaging-derived histogram parameters with the histological findings such as the cellular density. Further study with larger sample size that clarified the association among DK imaging-derived parameters, histological features, clinical stage and patient outcome would be more valuable. Fourth, we did not simultaneously enroll the conventional DW imaging as control. Further study that directly compare DK and DW imaging would be more valuable to confirm the superiority of DK imaging. Fifth, the test-retest repeatability of functional MR imaging was the hot point of clinical research, and associated with the wide application. However the test-retest repeatability of DK imaging was not assessed in our study, and this area was worth of more attention in future. Finally, the histogram analysis in our study was performed in a semi-quantitative way that depended on the diagnostic level of post-processing radiologists. In the future, the computer-aided analysis might be helpful to improve the reproducibility of imaging interpretation.

## MATERIALS AND METHODS

### Patients

This retrospective study was approved by our institutional review board, and the requirement to obtain written informed consent was waived due to the retrospective nature of our study. Between January 2015 and August 2016, a total of 58 consecutive NPC patients underwent DK imaging scan as a part of pretreatment MR imaging evaluation. We totally excluded 11 patients according to the following exclusion criterion: 1) biopsy before MR scan (*n* = 6); 2) the maximum diameter of the lesion was less than 1 cm (*n* = 2); 3) the image quality of DK image was not adequate for further analysis (*n* =3).

Finally, 47 patients (35 men and 12 women; mean age, 52 ± 14 years; range, 12 - 88 years) were enrolled in our study. The pathological diagnosis was all poorly differentiated squamous cell carcinoma. The TNM and AJCC stage were determined by two radiologists according to the 7th edition of the international Union against Cancer/American Joint Committee on Cancer (UICC/AJCC) staging system, based on all available clinical and imaging data (MR imaging of the nasopharynx and neck, computed tomography imaging of the thorax and abdomen or whole body [18F] fluorodeoxyglucose positron emission tomography/computed tomography) [2, 3]. In case of discrepancy, another senior radiologist would make the final decision. Detailed demographic and clinical information of the patients are summarized in Table 4.

**Table 4:** Clinical and demographic information of all the patients

Parameters	Results
Gender (male/female)	35:12
Age (years)	52 ± 14
Range (years)	12 − 88
T stage	
T1	18
T2	10
T3	7
T4	12
N stage	
N0	10
N1	21
N2	14
N3	2
M stage	
M0	44
M1	3
AJCC stage	
I	3
II	14
III	16
IV	14

Note: AJCC indicates American Joint Committee on Cancer.

### MR scan

MR examinations were performed by using a 3.0T MR system (Verio, Siemens Medical System, Erlangen, Germany) with a 12-channel head and neck coil. Conventional MR imaging scan ranged from the skull base to the suprasternal notch, and included the following sequence: 1) sagittal fat-suppressed T2-weighted image [repetition time (TR)/echo time (TE) = 4200/79 ms; slice number = 20; slice thickness = 4 mm without gap; field of view (FOV) = 240 mm; matrix = 384 × 384]; 2) sagittal T1-weighted image (TR/TE = 350/2.5 ms; slice number = 20; slice thickness = 4 mm without gap; FOV = 240mm; matrix = 384 × 384); 3) axial fat-suppressed T2-weighted image (TR/TE, 5930/95 ms; slice number = 40; slice thickness = 4 mm without gap; FOV = 230mm; matrix = 384 × 384); 4) axial T1-weighted image (TR/TE, 1460/10 ms; slice number = 40; slice thickness = 4 mm without gap; FOV = 230 mm; matrix = 384 × 384); 5) coronal fat-suppressed T2-weighted image (TR/TE, 4500/79 ms; slice number = 20; slice thickness = 4mm without gap; FOV = 240 mm; matrix = 384 × 384); and 6) coronal T1-weighted image (TR/TE, 712/9.5 ms; slice number = 20; slice thickness = 4mm without gap; FOV = 240 mm; matrix = 384 × 384). For the post-contrast MR scan, gadolinium-diethylene triamine pentaacetic acid (Magnevist, Bayer Schering Pharma AG, Berlin, Germany) was intravenously bolus injected at a dose of 0.1 mmol/kg of body weight, followed by a 20-mL bolus of saline. After the contrast injection, axial, coronal and sagittal T1-weighted images were acquired. The total scan time of conventional MR imaging was about 18 minutes and 30 seconds.

A readout-segmented echo-planar imaging (RS-EPI) sequence was used for DK imaging scan with 4 b values (0, 500, 1000 and 1500 s/mm^2^) in three orthogonal directions. The other imaging parameters were showed as follows: TR/TE, 4300/74ms; flip angle (FA), 180°; number of averages (NEX), 1; FOV, 230 mm; slice thickness, 4 mm without gap; matrix, 224 × 224. The total acquisition time of DK imaging was 4 minute 32 seconds.

### Image analysis

DK imaging data was processed using an in-house developed software (FireVoxel, CAI^2^R, New York University, NY) [17]. The relationship between signal intensity of DK image and b factors could be expressed by the following equation [7, 12]:

S_(b)_ = S_0_e^−bDapp+1/6(b^2^Dapp^2^Kapp)^

where S is the signal intensity, b is the b value (s/mm^2^), D_*app*_ is the apparent diffusion parameter of Gaussian distribution (10^–3^ mm^2^/s), and K_*app*_, a dimension-less parameter, is the apparent kurtosis coefficient.

ROIs were manually drawn on all the imaging sections of the primary lesions. The large necrotic, cystic, and hemorrhagic areas were excluded with reference to the T2-weighted and contrast-enhanced T1-weighted images. In tumors with muscle and skull base invasion, the involved skull base and muscle were excluded from ROIs. Histogram analysis of the DK imaging-derived parameters (D_*app*_ and K_*app*_) was performed by using PASW Statistics 18.0 (SPSS, Chicago, IL) [18]. Based on the whole-tumor ROIs, following histogram parameters were derived: 1) mean; 2) median; 3) kurtosis; 4) skewness. Kurtosis is the degree of the peakedness of the histogram distribution. The kurtosis values is equal to 3 when the histogram is Gaussian, > 3 with a sharper peak, and < 3 with a flatter top. Skewness is a measure of the degree of asymmetry of the histogram distribution. The skewness value is positive if the majority of the data is concentrated on the left of the histogram, and negative if the majority of data is concentrated on the right. We also measured two cumulative histogram parameters including the 10th and 90th percentiles of D_*app*_ and K_*app*_. The nth percentile was the point at which *n*% of the voxel values that form the histogram were found to the left [13].

All the ROIs placements were performed by two radiologists (reader 1: with 5 years of clinical experience in head and neck radiology; reader 2: with 4 years of clinical experience in head and neck radiology) who were blind to the study design. The measurement results of the two radiologists were used to assess the inter-reader reproducibility. The average of the two measurement results was used for further statistical analysis.

### Statistical analysis

Similar with previous studies [2, 3], due to the limited patient numbers in each separate disease stage, we divided the patients into low T-stage (T1/2) and high T-stage (T3/4) group, low N-stage (N0/1) and high N-stage (N2/3) group, and low AJCC-stage (I/II) and high AJCC-stage group (III/IV). Statistical analysis was not performed for the M stage due to the small patient number in the M1 stage (*n* = 3).

All continuous variables were expressed as mean ± standard deviation, and tested with Kolmogorov-Smirnov test for normally distributed analysis. Independent-sample *t* tests were used to compare the differences of DK imaging-derived histogram parameters between low and high T, N and AJCC stages, respectively. Then, multiple receiver operating characteristic (ROC) curves were performed to determine and compare the value of identified significant parameters in predicting high T, N and AJCC stage, respectively. The areas under curves (AUCs) were compared using the method of Delong *et al* [23]. Cut-off value was established by maximizing the Youden index (Youden index = sensitivity + specificity − 1).

The inter-reader reproducibility of the measurements of DK imaging-derived histogram parameters was evaluated by using intraclass correlation coefficient (ICC) with 95% confidence intervals [24]. The ICC value ranged from 0 to 1.00, with values closer to 1.00 representing better reproducibility. It was interpreted as follows: (< 0.40, poor; 0.41–0.60, moderate; 0.61–0.80, good; ≥ 0.81, excellent). All statistical analyses were performed by using statistical software (SPSS for windows, version 19.0, SPSS, Chicago, Ill; MedCalc for windows, version 9.0, MedCalc Software, Mariakerke, Belgium). Two-sided *P* values less than 0.05 were considered statistically different.

## CONCLUSIONS

Our study showed that DK imaging-derived histogram parameters were useful for characterizing NPC, and correlated well with the clinical stage of the NPC patients. D_*app-10th*_ was optimal for predicting the high N- and AJCC-stage, while K_*app-90th*_ was most powerful for predicting the high T-stage. Our results suggested that DK imaging-derived parameters could be a potential imaging marker for pretreatment staging, and DK imaging could be a useful adjunctive imaging technique for evaluating NPC before treatment.
